# Functional Brain Connectivity and Inhibitory Control in Older Adults: A Preliminary Study

**DOI:** 10.3389/fnagi.2022.763494

**Published:** 2022-03-14

**Authors:** Brandon M. Brewster, Marcia Smith Pasqualini, Laura E. Martin

**Affiliations:** ^1^School of Psychology and Cognitive Science, Avila University, Kansas City, MO, United States; ^2^Department of Psychology, Saint Louis University, St. Louis, MO, United States; ^3^Department of Population Health, University of Kansas Medical Center, Kansas City, KS, United States; ^4^Hoglund Biomedical Imaging Center, University of Kansas Medical Center, Kansas City, KS, United States; ^5^Cofrin Logan Center for Addiction Research and Treatment, University of Kansas, Lawrence, KS, United States

**Keywords:** dACC, Stroop, inhibitory control, resting-state, functional connectivity, salience network, fMRI, attention system

## Abstract

According to the inhibition deficit hypothesis, the ability to inhibit unwanted or irrelevant thoughts and behaviors decreases with age, which can have a significant impact on cognitive and emotional processing. However, studies examining inhibition and age have shown mixed results, with some studies finding a decrease in inhibitory control as individuals age, while others have found no relationship. The goal of this proof-of-concept study was to examine the underlying neural mechanisms that may explain why some older adults are better than others at inhibitory control by investigating the relationship between resting-state functional connectivity (rsFC) of the salience network, a network critical for detecting and focusing attention toward relevant stimuli while ignoring irrelevant information in the environment, and a behavioral measure of inhibitory control (Stroop Task interference score) in a sample of 65 healthy older individuals (ages 65+). Results revealed no direct effect of age on Stroop performance; however, there was an indirect effect of age on Stroop performance through rsFC. These results suggest that rsFC of the salience network may be an important factor to consider when it comes to understanding individual differences in inhibitory control behavior among older adults.

## Introduction

Normal aging influences many cognitive functions including attention, information processing, working memory, and inhibitory control (Damoiseaux et al., 2007). Inhibitory control plays an important role in maintaining focused attention and avoiding action tendencies that are overlearned (Rey-Mermet and Gade, 2017). Inefficient inhibitory control can lead to a compromised selective attention system, resulting in an intrusion of information into working memory that is not necessarily relevant for current tasks. These intrusions increase processing time and decrease recognition of relevant information (Kramer et al., 1994).

### Inhibitory Control and Aging

The age-related inhibition deficit hypothesis states that the ability to inhibit unwanted or irrelevant thoughts and behaviors decreases as individuals age (Hasher and Zacks, 1988). However, studies of this hypothesis have shown mixed results. Some studies demonstrate age-related inhibitory control deficits (Kramer et al., 1994; Andres et al., 2008), whereas others show no deficits (Salthouse, 2010; Sebastian et al., 2013) or even some improvements in inhibitory control among older adults compared to young adults (Madden and Gottlob, 1997; Fernandez-Duque and Black, 2006). A meta-analysis conducted by Rey-Mermet and Gade (2017) tested the inhibition deficit hypothesis to investigate whether this deficit was generalizable to older individuals, or if it was specific to certain tasks. Contrary to prior studies, results demonstrated that for most tasks, including the color Stroop task (Stroop, 1935), inhibitory control deficits were not present in older adults (Rey-Mermet and Gade, 2017). In the Stroop task, participants must selectively attend to and state the color the word is presented in, while engaging inhibitory control to inhibit the more automatic process of reading irrelevant color words presented (Spieler et al., 1996).

Stroop performance has been linked to specific neural correlates including activation in the anterior cingulate cortex (ACC; Duchek et al., 2013). Activation in the ACC has shown age-related increases when individuals were presented with incongruent color trials (Milham et al., 2002). Specifically, Milham et al. (2002) found that the presence of competing color information (having a color and color-word presented simultaneously, even if congruent) is enough to elicit an increase in neural activity within the ACC in older adults, while additional attentional demands of conflicting (incongruent) color information is necessary to evoke a significant increase in ACC activity within younger adults. Additionally, a between group-analysis revealed that only younger adults experienced this increase in ACC activity specific to conflicting incongruent trials. These results suggest that increased activation in the ACC among older adults may be related to an inhibitory control deficit irrespective of increasing cognitive demands.

As reported in previous studies, inhibitory control processes are executed by key frontal regions including the dorsal anterior cingulate cortex (dACC), anterior insula (AI), and dorsolateral prefrontal cortex (Milham et al., 2002; Duchek et al., 2013; Pan et al., 2018). The dACC has been identified as a brain region essential for inhibitory control (Petersen and Posner, 2012), evidenced by activation during cognitively demanding tasks that involve inhibition of competing irrelevant information, e.g., Stroop task (Bush et al., 2000). Similarly, Dosenbach et al. (2006) identified both the dACC and bilateral AI as brain regions that form the “core” of the human task-set system, a set of cognitive processes that are actively maintained during task performance, including inhibitory control. In addition, the dACC serves a prominent role in modulating attention, response selection, monitoring competition, complex motor control, motivation, novelty, error detection, working memory, and anticipation of cognitively demanding tasks (Bush et al., 2000) and is thought to be an essential hub of the salience network (Menon, 2011).

### The Salience Network

The salience network, anchored in the dACC and ventral AI, with nodes in the amygdala, hypothalamus, ventral striatum, and thalamus, was introduced by Seeley and colleagues in 2007. The salience network is a homeostatic system whose job is to detect biologically and cognitively salient external stimuli and internal events. This network also controls the subsequent switching between the default mode network and central-executive network to facilitate higher-order inhibitory control and working memory once salient stimuli are detected (Menon and Uddin, 2010; Menon, 2011). Overall, studies support the idea that the hubs of the salience network (AI and dACC) play key roles in the conscious integration of autonomic feedback and responses from internal goals and environmentally salient demands (Seeley, 2019).

Two recent studies provide evidence that extensive reorganization within and between the salience network and other neurocognitive networks is associated with age (Das et al., 2021; Snyder et al., 2021). These results suggest that functional connectivity between the salience network and other neurocognitive networks evolve throughout the life span (Snyder et al., 2021). Given that the dACC is a key node of the salience network (Seeley et al., 2007) and is critically involved in inhibitory control, the dACC may play a significant role in inhibitory control deficits related to aging.

### Functional Connectivity

Resting state functional connectivity (rsFC) identifies functionally linked brain regions that show a high level of temporal correlation during rest, as opposed to anatomically linked brain regions (Heuvel and Pol, 2010). The advantage of rsFC over task-based approaches is that rsFC can probe brain networks related to inhibitory control processes (e.g., salience network) without being dependent on task performance, which could be reflective of difficulties in other domains that are common in older adults, and not necessarily related to inhibitory control. Examining resting state networks, including the salience network, may elucidate the mixed findings in previous studies regarding inhibitory control ability during aging. It has been hypothesized that alterations in the functional connectivity between regions may be the underlying mechanisms related to observable, behavioral aging deficits (Damoiseaux et al., 2007). Functional connectivity within the salience network, specifically between the bilateral insula and ACC, has been shown to decrease with aging and may be related to cognitive decline (Onoda et al., 2012).

### Current Study

The goal of the current proof-of-concept study was to investigate the relationship between age, the rsFC within the salience network, and behavioral inhibitory control (i.e., the Stroop task performance). We hypothesized that the relationship between age and Stroop performance would be mediated by rsFC within the salience network: specifically, that age would be negatively correlated with rsFC, and that rsFC would be positively correlated with Stroop performance.

## Methods

### Participants

The current study consisted of analyses on deidentified behavioral and neuroimaging data from the University of Kansas Alzheimer’s Disease Research Center (KU ADRC) registry. Data were requested for participants who completed a resting-state functional magnetic resonance imaging (fMRI) scan and a behavioral measure of inhibitory control (Stroop) within 90 days of one another (*n* = 80). Of the 80 registry participants who met the inclusion criteria, 15 were excluded due to excessive motion censoring (greater than 20% of frames censored) leaving a total of 65 participants included in our analyses. The average time between cognitive testing and fMRI was 70.73 days (*SD* = 15.85). The age range was from 65 to 84 years old (*M* = 70.92, *SD* = 5.02). See Table 1 for additional demographic information. All participants were identified as having no cognitive impairment based on cognitive assessments conducted by the KU ADRC. This study was deemed non-human research by the University of Kansas Medical Center’s Human Subjects Committee due to the use of de-identified data.

**TABLE 1:** Demographics of the 65 participants included in this study who completed resting-state fMRI and the Stroop task within 90 days of one another and did not have excessive motion censoring.

Demographics table	Sex of participants		
	Male	Female	Both (total)
Total number of participants	18	47	65
**Age of participants at visit**			
Mean (standard deviation)	72.17 (5.46)	70.45 (4.82)	70.92 (5.02)
**Years of education**			
Mean (standard deviation)	17.06 (2.98)	15.70 (2.16)	16.08 (2.45)
**Hispanic**			
Yes	0	1	1
No	18	46	64
**Race**			
White	18	44	62
American Indian or Alaskan American	0	1	1
Black or African American	0	2	2
**Stroop calculated interference score**			
Mean (Standard Deviation)	30.89 (9.89)	35.68 (10.82)	34.35 (10.72)
**Days to MRI**			
Mean (standard deviation)	68.06 (17.31)	71.77 (15.33)	70.74 (15.85)

### Data Accessibility

The behavioral and neuroimaging data included in the following analyses is accessible upon request through the KU ADRC.^1^

### Stroop Task

Participants completed the Stroop task (Stroop, 1935) during an in-person testing appointment that included several other measures collected by the KU ADRC. The task had three separate parts. During the color naming task, participants were handed a card and then asked to look at the colored boxes and say the color of each box, going from left to right and top to bottom without skipping any, as quickly as they could. Next, for the word reading task, participants were handed a card with a list of color names printed in black ink. They were asked to read the words in the same order as before. The final part was the interference task. Participants were told that the card had color names written in ink that was a different color from the word that was written. They were told to state the color of ink each word was printed in, while ignoring the words, in the same order as for the other tasks. Right before they started it was reiterated that they must ignore the words and simply state the colors of the ink that they saw. Raw scores on interference, color-naming, and word-reading were the number of correct items within 45 s. To account for individual differences in speaking speed, we computed a Stroop Calculated Interference score by subtracting interference scores from color-naming scores (Scarpina and Tagini, 2017).

### MRI Data Acquisition and Analysis

MRI scanning was performed on a 3-Tesla Siemens Skyra scanner (Siemens, Erlangen, Germany). The anatomical scan consisted of a T1-weighted 3D MPRAGE sequence (TR/TE = 2,300/2.98 ms, matrix = 240 × 256, slice thickness = 1.2 mm, 176 slices) and was used for co-registration with the functional scan and spatial normalization. The resting-state functional scans consisted of a gradient echo blood oxygen level dependent (BOLD) scans acquired with eyes open (repetition time/echo time [TR/TE] = 3,000/25 ms, flip angle = 90°, matrix = 80 × 80, slice thickness = 3 mm, in-plane resolution = 2.9 × 2.9 mm, 105 data points).

MRI data preprocessing and statistical analyses took place in Analysis of Functional Neuroimages (AFNI; Cox, 1996) and implemented using afni_proc.py (Example 11). Anatomical data were skull stripped and normalized to standard Montreal Neurological Institute (MNI) space using non-linear warping with AFNI command @SS_warper and these parameters were applied to the functional data for spatial normalization. Segmentation of the anatomical datasets was performed in Freesurfer (Fischl, 2012) and used to estimate average signal in the ventricles and white matter. The first two volumes of the functional scans were removed, and transient signal spikes were removed from the data. Volumes were slice time corrected and co-registered to the minimum outlier within the run. Volumes where more than 5% of the brain voxels were considered outliers based on magnitude of deviations in the voxel’s time series was removed from the analysis. In addition, motion greater than 0.2 mm within a volume were censored and removed from the analysis. To reduce spurious variance in the analysis, nuisance variables included motion parameters, average ventricle signal, and average white matter signal. Using multiple regression, the predicted timecourse was constructed and subtracted from each voxel timecourse resulting in a residual timecourse for each voxel. The residual timecourse was then smoothed with a 4 mm FWHM Gaussian kernel, resampled to a 2.5 mm × 2.5 mm × 2.5 mm grid and transformed to MNI space. While current resting state methods do not require spatial smoothing, the current data were acquired at a resolution of 2.9 mm × 2.9 mm × 3 mm with a TR of 3 s. Therefore, consistent with the recommendations of Poldrack et al. (2011), Bijsterbosch et al. (2017), and Jenkinson and Chappell (2018), we applied spatial smoothing to significantly improve the signal to noise. We chose a 4 mm kernel, as small values of FWHM (typically 1.5–2 times the voxel size) are recommended to improve signal to noise, without losing the ability to find small regional activity (Bijsterbosch et al., 2017; Jenkinson and Chappell, 2018). In EPI datasets with high spatial resolution (2.5 mm or less isotropic voxels), high temporal resolution (TR below 1.5 s) and a time series length of at least 10 mins, smoothing is not always necessary (Bijsterbosch et al., 2017); however, for lower resolution datasets, such as ours with a TR of 3 s and a time series length of 5.25 mins (105 volumes), spatial smoothing is advantageous. In addition, data were pre-processed without spatial smoothing to provide a comprehensive analysis summary. Note the direction of the reported effects were similar; however, the *p*-values were larger in the unsmoothed analysis with reduced signal to noise (see “Supplementary Material”).

**FIGURE 1:**
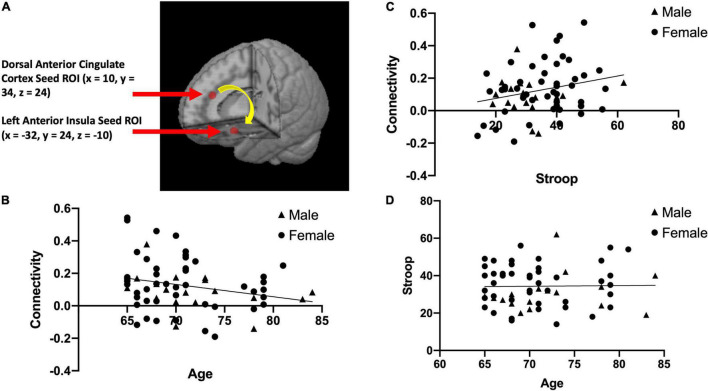
The two brain regions used in our resting state functional connectivity analysis of the salience network, the dorsal anterior cingulate cortex (*x* = 10, *y* = 34, *z* = 24) and left anterior insula (*x* = –32, *y* = 24, *z* = –10) adapted from Seeley et al. (2007) **(A)**. This panel also presents scatterplots between resting state functional connectivity (rsFC) of the salience network and Age; *r*(63) = –0.25, *p* = 0.049 **(B)**, between rsFC of the salience network and Stroop calculated interference score; *r*(63) = 0.24, *p* = 0.055 **(C)**, and between Stroop calculated interference score and Age; *r*(63) = 0.017, *p* = 0.89 **(D)**.

We used a seed-based approach to quantify functional connectivity between two nodes of the salience network (dACC and AI). This was done by creating spherical regions of interest (ROIs) with a 5 mm radius in the dACC and the left AI based on the salience network described in Seeley et al. (2007). We chose to focus a region of interest in the left AI for the primary analysis because of its uniqueness as part of both the salience network and executive control network (Seeley et al., 2007), making it essential for Stroop performance/inhibitory control (Grandjean et al., 2012). The center of the dACC ROI located at MNI coordinates *x* = 10, *y* = 34, *z* = 24 (Seeley et al., 2007; See Figure 1A) and the center of the left AI ROI located at MNI coordinates *x* = –32, *y* = 24, *z* = –10 (Seeley et al., 2007; See Figure 1A), were used to calculate rsFC within this network. We extracted the average time-series across the two regions of interest (dACC and left AI) for each participant, and computed Pearson correlations between the dACC and left AI. This correlation coefficient was then converted to Fisher *z*-transformed values for each participant.

### Exploratory Analyses

To further explore the salience network, we conducted seed-based exploratory parallel analyses by performing Pearson correlations on the rsFC of the dACC and right AI of the salience network. We correlated salience network scores with age, and with Stroop task performance scores, to investigate bilateralization of the salience network in relation to inhibitory control in the anterior insulas. We used MNI coordinates of the right AI, with the center of the ROI at *x* = 38, *y* = 26, *z* = −10, as previously used by Seeley et al. (2007), to identify and map the salience network.

In addition, we conducted a whole-brain analysis to identify brain regions where functional connectivity with the left AI correlated with Stroop performance. Whole brain analyses were corrected (voxelwise *p* < 0.01, *alpha* = 0.05).

### Analyses

To test our hypotheses, we investigated the relationships between our three main variables of interest: age, Fisher *z*-transformed resting-state functional connectivity (rsFC), and Stroop Calculated Interference score (Stroop). We first looked at Pearson product-moment correlations between these variables and corrected for multiple comparisons (Bonferroni). We then investigated whether rsFC mediates the relationship between age and Stroop by running a mediation analysis in SPSS using PROCESS with *Y* = Stroop, *X* = Age, M = rsFC and 1,000 bootstrap samples.

## Results

**FIGURE 2:**
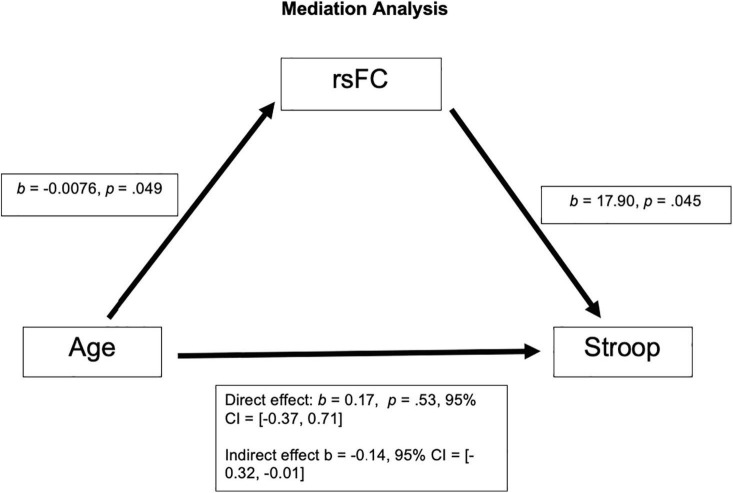
There was not a significant direct effect of Age on Stroop; however, there was a significant indirect effect of Age on Stroop through rsFC, *b* = −0.14, 95% CI = [−0.32, −0.01].

After correcting for multiple comparison (requiring *p* < 0.01 to indicate a significant relationship), Age and rsFC showed a pattern of negative correlation with each other [*r*(63) = −0.25, *p* = 0.049; See Figure 1B]. Stroop calculated interference score showed a pattern of positive correlation with rsFC, although not statistically significant [*r*(63) = 0.24, *p* = 0.055; see Figure 1C] and was not significantly correlated with age [*r*(63) = 0.017, *p* = 0.89; see Figure 1D]. The mediation analysis revealed that while there was no direct effect between age and Stroop (*b* = 0.17, 95% CI [−0.37, 0.71]), there was a significant indirect effect between age and Stroop through rsFC (*b* = −0.14, 95% CI [−0.32, −0.01]; See Figure 2). The 95% confidence interval does not cross zero, so we can assume it is a negative predictor (i.e., higher age is associated with poorer Stroop performance). Within this sample, we see that while rsFC was not significantly correlated with Age or Stroop performance when correcting for multiple comparisons, it did provide a significant indirect effect on the predictive value of age on Stroop.

Results of the exploratory analyses found that rsFC between right AI and dACC (*M* = 0.14, *SD* = 0.16) was not significantly correlated with Stroop calculated interference score [*r*(63) = −0.12, *p* = 0.36] or age, *r*(63) = −0.12, *p* = 0.36.

After corrections for multiple comparisons, results of the exploratory whole brain analysis did not find any regions that showed significant correlations between Stroop calculated interference score and functional connectivity with the dACC.

## Discussion

We found a pattern of a negative correlation between rsFC within the salience network (dACC and left AI) and age (*p* = 0.049), with rsFC of the salience network decreasing as age increases. Additionally, while the positive correlation between rsFC of the salience network (dACC and left AI) and Stroop performance failed to reach significance (*p* = 0.055), there was a significant indirect effect of age on Stroop performance through the rsFC of the salience network. These results indicate that as adults grow older, the rsFC between the left AI and dACC of the salience network decreases. Additionally, although this relationship did not reach statistical significance, the pattern suggests that older adults with greater connectivity within the salience network appear to perform better on the inhibitory control task (Stroop interference). Our results support previous findings (Onoda et al., 2012; Das et al., 2021; Snyder et al., 2021). These results also support the meta-analysis results (Rey-Mermet and Gade, 2017), which calls into question the inhibition deficit hypothesis, as we did not find a significant correlation between age and Stroop performance. While the meta-analysis investigated the correlation between age and behavioral measures of inhibitory control, our proof-of-concept approach builds on these prior studies by examining the relationship between functional connectivity within two nodes of the salience network and behavioral inhibitory control during healthy aging. In our sample, we found a significant indirect effect of rsFC on the relationship between age and Stroop, with no direct effect of age on Stroop.

The relationship between inhibitory control and aging is more complex than a simple linear relationship. There are many factors that influence inhibitory control as an individual ages; therefore, it is important to investigate the brain networks underlying inhibitory control, such as the salience network, rather than assuming that a decline in inhibitory control is strictly a factor of age. The current proof-of-concept study was a first step to inform future studies that will further enhance knowledge in the field regarding the relationship between functional connectivity within the salience network and inhibitory control in older adults using more sophisticated techniques. This could include classification approaches. For example, in studies examining children with autism, hyperconnectivity within the salience network was found to discriminate between children with autism and typically developing children (Uddin et al., 2013). In the same way, hypoconnectivity within the salience network during aging may be able to discriminate between individuals with and without inhibitory control deficits. Understanding these relationships will allow us to implement interventions that address specific age-related declines in cognitive processes earlier, to slow down the progression at a stage when interventions are most beneficial.

### Limitations

A key limitation is that we used secondary data and could not control what data were collected and when they were collected. For example, the Stroop task and rsFC connectivity scan were not conducted on the same day for participants. In addition, our sample included primarily white female volunteers with a high level of education, which may limit the generalizability of the data to the larger population. However, visual inspection of the data (see Figure 1) suggest that sex may not differentially impact the results. Another limitation is that our participants were all age 65 or older; with this limited range, it is difficult to fully evaluate the role that age plays in Stroop performance and rsFC. In addition, the correlation coefficients indicated small to medium effect sizes and were not statistically significant following corrections for multiple comparisons. Finally, the proof-of-concept analysis approach was limited to examining functional connectivity between two nodes of the salience network. Future studies should include network based analyses to fully elucidate the role of functional connectivity within and between networks in inhibitory control deficits that may occur during aging.

## Conclusion

We used a hypothesis-driven approach to look specifically at the salience network, identifying key seed regions on which to focus our analyses. Our results yielded a pattern of a negative correlation between rsFC of the salience network and age and positive correlation between rsFC and inhibitory control. Additionally, age was not significantly associated with inhibitory control; however, there was an indirect effect of age on Stroop performance through rsFC of the salience network. Overall, these preliminary results suggest that the relationship between inhibitory control and age may be driven by age related effects on the salience network. Future cognitive interventions should help increase inhibitory control in aging individuals by targeting the salience network. If effective, this approach may allow older adults to make informed decisions and perform cognitively demanding tasks while inhibiting irrelevant information.

## Data Availability Statement

The datasets analyzed in this study may be requested from the University of Kansas Medical Center. https://redcap.kumc.edu/surveys/?s=wQMXHa. Requests to access these datasets should be directed to LM, lmartin2@kumc.edu.

## Author Contributions

BB and LM were responsible for developing the study concept and design, acquired the data from the KU ADC through a research proposal, performed data processing and analysis for the experiment, and drafted the manuscript. MP provided feedback on the study design and assisted with interpretation of findings, critically reviewed the content and approved the final version for publication. All authors contributed to the article and approved the submitted version.

## Conflict of Interest

The authors declare that the research was conducted in the absence of any commercial or financial relationships that could be construed as a potential conflict of interest.

## Publisher’s Note

All claims expressed in this article are solely those of the authors and do not necessarily represent those of their affiliated organizations, or those of the publisher, the editors and the reviewers. Any product that may be evaluated in this article, or claim that may be made by its manufacturer, is not guaranteed or endorsed by the publisher.

## Footnotes

1
https://www.kumc.edu/research/alzheimers-disease-research-center
