# Supplementary material for: Functional Brain Connectivity and Inhibitory Control in Older Adults: A Preliminary Study
Source: Front Aging Neurosci. 2022 Mar 14;14:763494. doi: 10.3389/fnagi.2022.763494 (PMC8964462; doi:10.3389/fnagi.2022.763494)
Supplement: Supplementary file 1 [file Data_Sheet_1.DOCX]

**Supplementary Materials**

**MRI Analysis Without Smoothing**

Pre-processing: MRI data preprocessing and statistical analyses took place in Analysis of Functional Neuroimages (AFNI; Cox, 1996) and implemented using afni_proc.py (Example 11). Data preprocessing included all of the steps described in the manuscript how no spatial smoothing was applied.

Analyses: Analyses were performed on the data with no spatial smoothing as described in the manuscript using a seed-based approach to quantify functional connectivity between two nodes of the salience network (i.e., dACC and anterior insula). We extracted the average time-series across the two regions of interest (dACC and left AI) for each participant, and computed Pearson correlations between the dACC and left AI. This correlation coefficient was then converted to Fisher *z*-transformed values for each participant. Exploratory analyses examined connectivity between the dACC and right AI as well as a whole-brain analysis to identify brain regions where functional connectivity with the left AI correlated with Stroop performance. Whole brain analyses were corrected (voxelwise *p* < 0.01, *alpha* = 0.05)

Results: Age and rsFC showed a pattern of negative correlation with each other (*r*(63) = -.19, *p* = .13). Stroop calculated interference score showed a pattern of positive correlation with rsFC, although not statistically significant (*r*(63) = .19, *p* = .13). The mediation analysis revealed no direct or indirect effect between age and Stroop. Results of the exploratory analyses found that rsFC between right AI and dACC showed a pattern of a negative correlation with Stroop calculated interference score, (*r*(63) = -.20, *p* = .12) and no significant correlation with age, *r*(63) = -.10, *p* = .44. After corrections for multiple comparisons, results of the exploratory whole brain analysis did not find any regions that showed significant correlations between Stroop calculated interference score and functional connectivity with the dACC.

**Subsample Analysis**

A limitation of the primary analysis was an imbalance of male to female participants (72% female). To better understand the potential impact of this imbalance on the data we have analyzed the data with equal numbers of male and female participants by randomly selecting 18 female participants. Age and Stroop scores in the subsample of females (Age: Mean = 70.11, SD = 4.74, Stroop: Mean = 36.33, SD = 11.04) were similar to those in the full female sample (Age: Mean = 70.45, SD = 4.82, Stroop: Mean = 35.68, SD = 10.82). Primary analyses demonstrated a similar pattern to the full sample. Age and rsFC showed a pattern of negative correlation with each other (*r*(34) = -.22, *p* = .20) and Stroop calculated interference score showed a pattern of positive correlation with rsFC (*r*(34) = .27, *p* = .12), although not statistically significant.
